# Scar Rupture in Early Puerperium: A Case Report

**DOI:** 10.31729/jnma.7346

**Published:** 2022-05-31

**Authors:** Jyoti Dahal, Rachana Saha

**Affiliations:** 1 Department of Obstetrics and Gynecology, Kathmandu Medical College and Teaching Hospital, Sinamangal, Kathmandu, Nepal

**Keywords:** *caesarean section*, *case reports*, *postpartum*, *rupture*, *scar*

## Abstract

Uterine scar dehiscence or rupture is a rare but potentially life-threatening complication of caesarean delivery. It is the opening of the uterine incision line that can lead to postpartum haemorrhage, pelvic hematoma, pelvic abscess, endomyometritis, generalised or localised peritonitis and sepsis. Here we report a case of a 25 years old female who presented with puerperal pyrexia. Investigations revealed uterine scar rupture with a uterovesical collection. The case was managed conservatively with intravenous antibiotics, an intracervical Foley's catheter and a gonadotropin-releasing hormone agonist.

## INTRODUCTION

The rate of caesarean sections is increasing worldwide. Due to this, infrequent complications of Lower Segment Caesarean Section (LSCS) have been encountered. Uterine scar dehiscence or rupture following caesarean section is one of the rare complications. It is the opening of the uterine incision line and the frequency is between 0.06-3.8%.^1,2^ It can lead to postpartum haemorrhage, pelvic hematoma, pelvic abscess, endomyometritis, generalised or localised peritonitis and sepsis.^3^ Published cases have shown that symptoms can be seen as early as a week following caesarean section to 3 months. Management includes a conservative approach, laparotomy with reapproximation of the scar margins and hysterectomy. We hereby report a case of postcesarean uterine scar rupture which was managed conservatively with cervical catheter placement and intravenous antibiotics.

## CASE REPORT

A 25-year-old with obstetric index P1L1 woman was referred to our hospital for the care of puerperal pyrexia from the periphery of Eastern Nepal. The patient had undergone Emergency LSCS 10 days prior to admission at a hospital in Okhaldhunga and was admitted via emergency with chief complaints of fever for 9 days. LSCS was done for non-progression of labour following 20 hours of labour. She had rupture of membranes hours prior to presentation at the hospital and induction of labour was done after hours.

Her temperature was recorded to be 102°F on her first Postoperative Day (POD). She was diagnosed with a urinary tract infection and was started on Ceftriaxone. The fever did not subside in a week and she was referred to a higher centre for diagnosis and further management.

**Figure 1:**
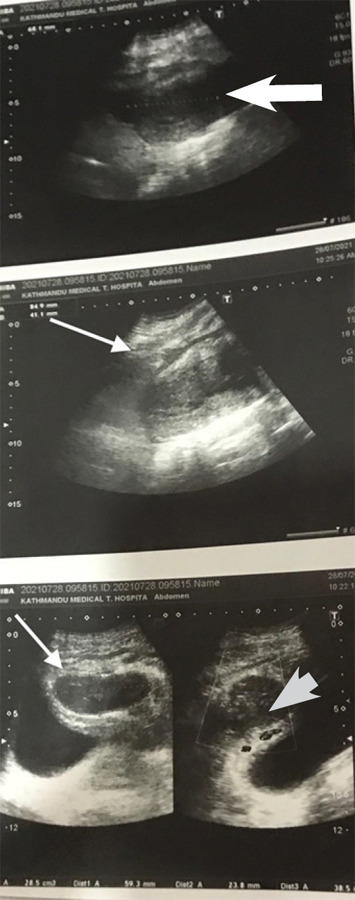
Ultrasonographic images showing uterus and uterovesical collection.

At the time of presentation, her general condition was good. She had a temperature of 100.9°F. Uterine involution was normal and the incision site of the skin was intact. Anterior abdominal wall oedema was present. The patient's investigations showed raised white blood cell count, predominantly neutrophils. And a routine microscopic examination of urine showed plenty of pus cells. Her biochemical parameters were within normal limits. Ultrasonography (USG) showed a defect in the lower anterior myometrial wall measuring 19.4 mm with heterogeneous collections measuring 28.5 ml within the uterine cavity communicating anteriorly forming a localised collection in the uterovesical pouch. The moderate intraperitoneal collection was noted (Figure 1).

Magnetic Resonance Imaging (MRI) was done the next day which showed a defect of 7 mm seen within the anterior uterine wall which is in direct continuity with a heterogeneous collection measuring 34 × 46 mm anterior to the uterus within the pelvic cavity. It also showed a moderate amount of oedema within the subcutaneous tissues of the abdominal wall.

**Figure 2:**
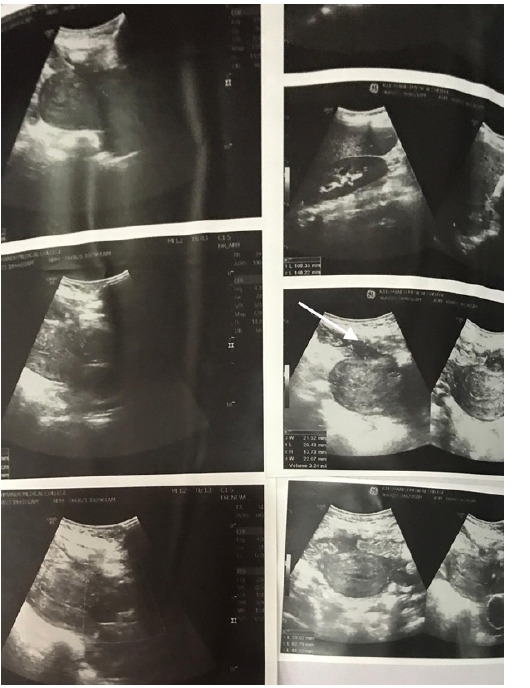
Ultrasonographic images after 9 days showing minimal uterovesical collection.

The patient was started on piperacillin, tazobactam and clindamycin. However, she persistently had a fever. The skin incision was opened and serosanguinous discharge was obtained which was sent for culture and sensitivity and the dressing was done daily. Intracervical Foley catheter (16 Fr) was placed to drain intrauterine collection 3 days following admission. Around 5 ml of the collection was drained and sent for culture and sensitivity. *Acinetobacter baumanii* was isolated in wound swab culture and sensitivity which was sensitive to meropenem and antibiotics were changed accordingly. Other cultures didn't show the growth of any organisms. The fever subsided and a repeat USG done 9 days later showed a defect of 5 mm in the lower anterior myometrial wall and minimal heterogeneous collection of 3.24 ml anterior to the uterus (Figure 2).

The patient was afebrile and the intracervical Foley catheter was kept for 10 days and removed. The total collection was 20 ml. The skin incision was resutured on 21^st^ POD. She was started on Gonadotropin-Releasing Hormone (GnRH) analogue (Injection Leuprolide) 3.75 mg intramuscular monthly for 3 months. USG done 17 days following admission only revealed inflammatory changes anterior to the uterus. No defects and collections were visualised. The patient was followed after 3 months and USG was done which revealed closure of the defect.

## DISCUSSION

Postpartum uterine scar dehiscence or rupture is a rare but potentially life-threatening condition characterised by the opening of the uterine incision. The important risk factors include diabetes, emergency surgery, surgical technique, infection, haematoma on the uterine incision line, uterovesical hematoma, previous caesarean section, classical caesarean section, abnormal placentation and inappropriate oxytocin administration.^1^ A study reported a 25 years old woman with uterine scar dehiscence 1 week following emergency caesarean section who presented with LSCS wound infection and was managed with exploratory laparotomy and reapproximation of the uterine defect with interrupted sutures.^4^

A study reported three cases of post-cesarean uterine scar dehiscence managed conservatively. All three cases presented 1-2 weeks following caesarean section with complaints of abdominal pain and purulent vaginal discharge. All cases were managed with intravenous antibiotics and were discharged within 2-4 weeks.^1^

A case of 27 years old with previous two LSCS with secondary Postpartum Haemorrhage (PPH) following uterine scar dehiscence following elective caesarean section 10 weeks back and managed with emergency laparotomy with a total abdominal hysterectomy and blood transfusion has been reported.^5^ A case of 35 years female who presented on the fifth POD following LSCS with pain and abdominal distention has also been published. Computed Tomography (CT) scan showed dehiscence of lower uterine caesarean section incision.

The case was managed with emergency laparotomy with hysterectomy.^6^ Any imaging modalities such as ultrasonography, magnetic resonance imaging, and computed tomography can be used for the diagnosis of post-cesarean scar rupture.^1^

Exploratory laparotomy must be considered the most important tool for diagnosis and treatment for uterine scar dehiscence or rupture. A conservative approach with reapproximation of healthy margins can be considered. However, in case of marked wound infection, endomyometritis, and/or intraabdominal abscess, a hysterectomy must be considered.^4^ Conservative approach with intravenous antibiotics and drainage of the pelvic collection can be considered in stable patients with no evidence of active haemorrhage or severe infection.^1^

This case report highlights a rare but important complication of caesarean section. As this condition is rarely encountered, there are no published guidelines regarding the management of the condition. Intracervical catheter placement and postponement of menstruation by GnRH analogue were different techniques employed in this case. A high degree of suspicion with appropriate investigations will help in identifying postpartum uterine wound dehiscence or rupture and its timely management will prevent maternal mortality and morbidity.
